# Supplementary material for: From sequence to enzyme mechanism using multi-label machine learning
Source: BMC Bioinformatics. 2014 May 19;15:150. doi: 10.1186/1471-2105-15-150 (PMC4229970; doi:10.1186/1471-2105-15-150)
Supplement: Additional file 2 — Java code of ml2db. Additional file ml2db_code.tar.gz contains the Java source code to run the multi-label machine learning experiments and save the results to database. The code’s Javadoc is included. [file 1471-2105-15-150-S2.zip › additional file 2/ml2db/ml2db/doc/mulan/classifier/lazy/MLkNN.html]

MLkNN


JavaScript is disabled on your browser.


- Overview
- Package
- Class
- Use
- Tree
- Deprecated
- Index
- Help

- Prev Class
- Next Class

- Frames
- No Frames

- All Classes

- Summary:
- Nested |
- Field |
- Constr |
- Method

- Detail:
- Field |
- Constr |
- Method


mulan.classifier.lazy

## Class MLkNN

- java.lang.Object
- - mulan.classifier.MultiLabelLearnerBase
  - - mulan.classifier.lazy.MultiLabelKNN
    - - mulan.classifier.lazy.MLkNN

- All Implemented Interfaces:
  :   java.io.Serializable, MultiLabelLearner, weka.core.TechnicalInformationHandler

  ---

    

  ```
  public class MLkNN
  extends MultiLabelKNN
  ```

  Class implementing the ML-kNN (Multi-Label k Nearest Neighbours) algorithm.

  Version:
  :   2012.07.16

  Author:
  :   Eleftherios Spyromitros-Xioufis

  See Also:
  :   Serialized Form

- - ### Field Summary

    - ### Fields inherited from class mulan.classifier.lazy.MultiLabelKNN

      `WEIGHT_INVERSE, WEIGHT_NONE, WEIGHT_SIMILARITY`
  - ### Constructor Summary

    Constructors

    Constructor and Description

    `MLkNN()`

    The default constructor

    `MLkNN(int numOfNeighbors,
    double smooth)`
  - ### Method Summary

    Methods

    | Modifier and Type | Method and Description |
    | --- | --- |
    | `weka.core.TechnicalInformation` | `getTechnicalInformation()` Returns an instance of a TechnicalInformation object, containing detailed information about the technical background of this class, e.g., paper reference or book this class is based on. |
    | `java.lang.String` | `globalInfo()` Returns a string describing the multi-label learner. |

    - ### Methods inherited from class mulan.classifier.lazy.MultiLabelKNN

      `isUpdatable, setDfunc, setDistanceWeighting`
    - ### Methods inherited from class mulan.classifier.MultiLabelLearnerBase

      `build, getDebug, makeCopy, makePrediction, setDebug`
    - ### Methods inherited from class java.lang.Object

      `equals, getClass, hashCode, notify, notifyAll, toString, wait, wait, wait`

- - ### Constructor Detail


    - #### MLkNN

      ```
      public MLkNN(int numOfNeighbors,
           double smooth)
      ```

      Parameters:
      :   `numOfNeighbors` - : the number of neighbors
      :   `smooth` - : the smoothing factor


    - #### MLkNN

      ```
      public MLkNN()
      ```

      The default constructor
  - ### Method Detail


    - #### globalInfo

      ```
      public java.lang.String globalInfo()
      ```

      **Description copied from class: `MultiLabelLearnerBase`**

      Returns a string describing the multi-label learner.

      **Specified by:**
      :   `globalInfo` in class `MultiLabelLearnerBase`

      Returns:
      :   a description suitable for displaying in a future gui


    - #### getTechnicalInformation

      ```
      public weka.core.TechnicalInformation getTechnicalInformation()
      ```

      **Description copied from class: `MultiLabelLearnerBase`**

      Returns an instance of a TechnicalInformation object, containing detailed
      information about the technical background of this class, e.g., paper
      reference or book this class is based on.

      **Specified by:**
      :   `getTechnicalInformation` in interface `weka.core.TechnicalInformationHandler`

      **Specified by:**
      :   `getTechnicalInformation` in class `MultiLabelLearnerBase`

      Returns:
      :   the technical information about this class


- Overview
- Package
- Class
- Use
- Tree
- Deprecated
- Index
- Help

- Prev Class
- Next Class

- Frames
- No Frames

- All Classes

- Summary:
- Nested |
- Field |
- Constr |
- Method

- Detail:
- Field |
- Constr |
- Method
